# Clinical correlation of nonalcoholic fatty liver disease in a Chinese taxi drivers population in Taiwan: Experience at a teaching hospital

**DOI:** 10.1186/1756-0500-4-315

**Published:** 2011-08-31

**Authors:** Tao-Hsin Tung, Tsung-Hung Chang, Wei-Hsiu Chiu, Tzu-Han Lin, Hui-Chuan Shih, Ming-Huei Chang, Jorn-Hon Liu

**Affiliations:** 1 Cheng-Hsin General Hospital, Taipei, Taiwan, ROC; 2 Department of Public Health, College of Medicine, Fu-Jen Catholic University, Taipei, Taiwan, ROC; 3 Department of Nursing, Kang-Ning Junior College of Medical Care and Management, Taipei, Taiwan, ROC; 4 Kaohsiung Armed Forces General Hospital, Kaohsiung, Taiwan, ROC; 5 Department of Biomedical Imaging and Radiological Sciences, School of Biomedical Science and Engineering, National Yang-Ming University, Taipei, Taiwan, ROC; 6 Central Clinic and Hospital, Taipei, Taiwan, ROC; 7 Department of Obstetrics and Gynecology, Chung Shan Hospital, Taipei, Taiwan, ROC; 8 Faculty of Medicine, School of Medicine, National Yang-Ming University, Taipei, Taiwan, ROC

**Keywords:** nonalcoholic fatty liver disease, prevalence, taxi drivers, gender difference

## Abstract

**Background:**

To explore any gender-related differences in the prevalence of conditions-associated with non-alcoholic fatty liver disease (NAFLD) among Taiwanese taxi drivers in Taipei, Taiwan.

**Methods:**

We studied 1635 healthy taxi drivers (1541 males and 94 females) who volunteered for physical check-ups in 2006. Blood samples and ultrasound fatty liver sonography results were collected.

**Results:**

The prevalence of NAFLD was 66.4% and revealed no statistically significant decrease with increasing age (p = 0.58). Males exhibited a greater prevalence of NAFLD than did females (67.5% vs 47.9%, p < 0.0001). Gender-related differences for associated factors were found. For males, hypertension, hyperuricemia, higher AST, higher ALT, hypertriglyceridemia, and higher fasting plasma glucose were significantly related to NAFLD. These conditions were not sigfinicantly related to NAFLD in females.

**Conclusion:**

Several gender-related differences were noted for NAFLD among Taiwanese taxi drivers.

## Background

Non-alcoholic fatty liver disease (NAFLD) is a clinic-pathological diagnosis characterized by macrovesicular steatosis in hepatocytes and metabolic stress-related liver disorder, occurring in the absence of other causes of chronic liver disease [1]. It includes a wide spectrum of liver diseases, ranging from pure fatty liver disease, which is usually a benign and nonprogressive condition, to nonalcoholic steatohepatitis (NASH), which may eventually progress to liver cirrhosis, portal hypertension, and hepatocellular carcinoma [2]. Epidemiological studies of fatty liver disease showed some important risk factors. In China, along with the improved living standard and increased alcohol consumption, population-based surveys have revealed that fatty liver has become a serious public health problem [3,4]. In addition, the steadily increasing morbidity of obesity, coupled with type 2 diabetes, dyslipidemia, hypertension, and ultimately the metabolic syndrome has also put a large proportion of the Chinese population at risk[5]. From the preventive medicine viewpoint, early detection of this liver disorder by screening followed by appropriate intervention may offer a practical means for the prevention of condition-associated hepatocellular damage.

In Taiwan, the "taxi" industry is a lightly restricted vocational. A person can be a taxi driver if he or she has an occupational driver's license, practice registration, and a taxi car. It is often the choice of people with a lower education and a lack of economic, social, or human capital. A survey revealed that more than 90% of taxi drivers were male, 70% were between 40 and 59 years of age, and 50% had a junior high school education or below. The average working time was 10.3 hours a day, 6.2 days a week. They spent 3.5 hours waiting for passengers. Due to their being alienated from a workplace environment, repetition, monotony, boredom, a low sense of control over their work, and the stress of coping with various traffic conditions, they had high physical and mental health risks.

To the best of our knowledge, few studies have explored the epidemiologic information of NAFLD among taxi drivers in Taiwan. Knowing the background morbidity of NAFLD in this highly pressured population is important. In addition, the complete spectrum of demographic and biological markers which may be related to NAFLD should also be explored. Some uncertainty still exists regarding the prevalence of and the associated risk factors for NAFLD, in terms of gender differences. This study was designed to clarify these relationships.

## Methods

### Data resource and data collection

Company-employed or self-employed taxi drivers were the targeted population. This cross-sectional study was conducted between January 2006 and December 2006 with 1635 taxi drivers (1541 males and 94 females) voluntarily admitted to one teaching hospital in northern Taiwan for an annual physical check-up. Blood samples and ultrasound sonography results were collected. Clinical nurses drew fasting blood samples via venipuncture from the study participants. Overnight-fasting serum and plasma samples (from whole blood preserved with EDTA and NaF) were kept frozen (-20°C) until ready for analysis. Disease or conditions were defined by certain standards as follows: high fasting plasma glucose (FPG)≥110 mg/dl, obesity: a body mass index (BMI)≥27 Kg/m^2^, hypertension: a high systolic blood pressure (SBP) ≥140 mmHg or high diastolic blood pressure (DBP) ≥90 mmHg, hypercholesterolemia (≥200 mg/dL), hypertriglyceridemia (≥200 mg/dL), and hyperuricemia (≥7 mg/dL for males or ≥6 mg/dL for females). A serum ALT or AST level ≥40 U/L was classified as elevated [6].

### Ultrasound examination

Hepatic ultrasonography for all study participants was performed by the well-trained ultrasonographist using a Toshiba Nemio (SSA-550A) ultrasound probe. The ultrasonographic criteria which were used to diagnose a fatty liver included liver and kidney echo discrepancy, presence of an increased liver echogenicity, echo penetration into the deep portion of the liver, and clarity of the liver blood vessel structures [7]. The definition of NAFLD remains clinicopathological with well-defined criteria of the patterns of liver injury [8,9]. For the operational purposes of this study, the definition of NAFLD used surrogate indicators of the disease, that is, hepatic steatosis in persons who have negative serologic tests for viral hepatitis, autoimmune liver disease, and congenital causes of chronic hepatitis[8]. All study subjects who were diagnosed as NAFLD at outpatient department by ultrasound and without history of chronic hepatitis and alcohol consumption were enrolled the study. In addition, the degree of NAFLD on ultrasonography was divided into mild and severe cases.

### Interobserver reliability in ultrasound sonography

To establish a consistent diagnosis of NAFLD between specialists, the Kappa statistic was used to assess the agreement of inter-observer reliability among specialists in this study. A pilot study was performed using 100 randomly selected healthy subjects other than the study participants. For inter-observer reliability, the Kappa value for diagnosis of NAFLDS between specialists was 0.78 (95%CI: 0.70-0.85).

### Statistical analysis

Statistical analysis was performed using SAS for Windows, (SAS version 9.1; SAS Institute Inc., Cary, NC, USA). A p-value of <0.05 was considered to represent a statistically significant difference between the two test populations. For univariate analysis, the two-sample, independent t-test method was adopted to assess differences in the mean value of continuous variables between subjects with and without NAFLD. Crude and adjusted odds ratios (adjustment for gender and age) were estimated and 95% confidence intervals were used. Multiple logistic regression was also performed to investigate the independence of risk factors associated with the prevalence of NAFLD.

## Results

Table 1 shows, the overall prevalence of NAFLD for the screened population was 66.4%. The χ^2 ^trend test revealed no statistically significant decrease with increasing subject age (p = 0.58). The prevalence of NAFLD for males proved to be significantly greater than it was for females (respectively, 67.5% vs 47.9%, χ^2 ^test = 16.44, p < 0.001). In addition, after stratifying the data by age into one of four broad (age) groups, males exhibited a more-pronounced prevalence of NAFLD for all age groups than females.

**Table 1:** The gender and age specific prevalence of NAFLD among screened taxi drivers (n = 1635)

	Male(n = 1541)				Female(n = 94)					Total(n = 1635)		
			
Age (yrs)	Screened No	NAFLD No	Prevalence (%)	p-value for trend test	Screened No	NAFLD No	Prevalence (%)	p-valuefor trend test	Screened No	NAFLD No	Prevalence (%)	p-value for trend test
20-39	172	112	65.1		7	2	28.6		179	114	63.7	
40-49	538	381	70.8	0.24	33	10	30.3	0.01	571	391	68.5	0.58
50-59	696	457	65.7		50	32	64.0		746	489	65.5	
≥60	135	90	66.7		4	1	25.0		139	91	65.5	
Total	1541	1040	67.5		94	45	47.9		1635	1085	66.4	

The χ^2 ^trend test revealed a significant positive relationship with age (p = 0.01) for female study subjects but not for males (p = 0.24).

Table 2 shows the results of the comparison of a variety of test characteristics and their potential association with the specific (NAFLD) class value (yes or no) for the taxi drivers. The two-sample independent *t*-test found that SBP, DBP, BMI, uric acid, AST, ALT, total cholesterol, triglyceride, and FPG were associated with NAFLD. In addition to SBP, DBP, BMI, uric acid, AST, ALT, total cholesterol, and triglyceride were significantly associated with NAFLD in both in males and in females. ALP was significantly related to NAFLD only for female taxi driver subjects. FPG was significantly related to NAFLD in males not in females. (Table 3)

**Table 2:** Comparisons of characteristics of NAFLD among screened taxi drivers

	NAFLD			
	
	No (n = 550)	Yes (n = 1085)	Total (n = 1635)	p-value
				
Variables	Mean ± SD	Mean ± SD	Mean ± SD	
Age(yr)	49.71 ± 7.82	49.57 ± 7.60	49.62 ± 7.67	0.73
SBP(mmHg)	120.93 ± 15.43	127.81 ± 15.48	125.49 ± 15.80	<0.001
DBP(mmHg)	76.58 ± 10.18	81.82 ± 10.36	80.06 ± 10.59	<0.001
BMI(Kg/m^2^)	23.28 ± 2.77	26.64 ± 3.41	25.51 ± 3.58	<0.001
Uric acid(mg/dl)	5.97 ± 1.32	6.85 ± 1.44	6.55 ± 1.46	<0.001
AST(U/L)	22.99 ± 13.83	26.70 ± 17.49	25.45 ± 16.44	<0.001
ALT(U/L)	26.95 ± 27.73	38.32 ± 32.56	34.50 ± 31.47	<0.001
Total cholesterol (mg/dl)	202.25 ± 35.84	208.63 ± 35.49	206.48 ± 35.73	<0.001
Triglyceride (mg/dl)	115.71 ± 85.18	192.67 ± 185.44	166.78 ± 163.02	<0.001
Fasting plasma glucose (mg/dl)	90.47 ± 15.12	97.48 ± 28.11	95.12 ± 24.74	<0.001
ALP(U/L)	150.07 ± 38.99	153.53 ± 37.77	152.36 ± 38.21	0.08

**Table 3:** Comparisons of characteristics of NAFLD stratified by gender among screened taxi drivers

	Male(n = 1541)			Female(n = 94)		
				
	**With NAFLD**	**Without NAFLD**	p-value	**With NAFLD**	**Without NAFLD**	p-value
	Mean ± SD	Mean ± SD		Mean ± SD	Mean ± SD	
Age(year)	49.51 ± 7.68	49.81 ± 7.84	0.48	50.89 ± 5.35	48.67 ± 7.59	0.10
SBP(mmHg)	128.17 ± 15.45	121.97 ± 15.14	<0.001	119.40 ± 13.81	110.22 ± 119.40	0.002
DBP(mmHg)	82.00 ± 10.41	77.22 ± 10.10	<0.001	77.60 ± 8.13	70.02 ± 8.48	<0.001
BMI(kg/m^2^)	26.67 ± 3.40	23.38 ± 2.80	<0.001	25.88 ± 3.44	22.23 ± 2.12	<0.001
Uric acid(mg/dl)	6.90 ± 1.43	6.10 ± 1.29	<0.001	5.72 ± 1.20	4.66 ± 0.86	<0.001
AST(mg/dl)	26.47 ± 16.97	23.30 ± 14.34	<0.001	31.96 ± 26.57	19.84 ± 5.93	0.004
ALT(mg/dl)	38.36 ± 32.36	27.76 ± 28.74	<0.001	37.33 ± 37.13	18.71 ± 10.82	0.002
ALP (U/L)	153.06 ± 37.24	151.63 ± 38.24	0.490	164.36 ± 47.73	134.06 ± 43.26	0.002
Total cholesterol (mg/dl)	208.36 ± 35.60	203.29 ± 35.42	0.009	214.96 ± 32.45	191.55 ± 38.69	0.002
Triglyceride (mg/dl)	194.62 ± 188.56	118.23 ± 87.73	<0.001	147.56 ± 73.63	89.94 ± 45.52	<0.001
Fasting plasma glucose (mg/dl)	97.64 ± 28.55	90.51 ± 15.32	<0.001	93.89 ± 13.93	90.08 ± 13.10	0.18

Table 4 presents the crude and adjusted odds ratios for the association between certain relevant associated risk factors and NAFLD. Subjects with NAFLD had a more-pronounced prevalence of hypertension (adjusted OR = 2.26, 95%CI: 1.69-3.03), a higher BMI (adjusted OR = 6.88, 95%CI: 5.46-8.66), more hyperuricemia (adjusted OR = 3.08, 95%CI: 1.95-4.88), higher AST (adjusted OR = 2.04, 95%CI: 1.33-3.13), higher ALT (adjusted OR = 3.79, 95%CI: 2.82-5.10), more hypercholesterolemia (adjusted OR = 1.31, 95%CI: 1.07-1.81), more hypertriglyceridemia (adjusted OR = 4.16, 95%CI: 3.01-5.75), and higher FPG (adjusted OR = 3.11, 95%CI: 1.98-4.89) than subjects without NAFLD, subsequent to adjustment for gender and age.

**Table 4:** Crude and adjusted odds ratio of associated factors for NAFLD among screened taxi drivers

Item		With NAFLD (n = 1085)	Without NAFLD (n = 550)	Crude odds ratio	Adjusted odds ratio ^1^
Gender	female	45	49	2.26	---
	male	1040	501	(1.49-3.44)	---
Age	20-39	114	65	1.00	---
	40-49	391	180	0.93 (0.58-1.47)	---
	50-59	489	257	1.15 (0.69-1.47)	
	≥60	91	48	1.00 (0.77-1.70)	---
Hypertension	yes	824	487	2.28	2.26
	no	261	67	(1.71-3.06)	(1.69-3.03)
Higher BMI	yes	865	200	6.96	6.88
	no	217	349	(5.53-8.745)	(5.46-8.66)
Hyperuricemia	yes	956	527	3.07	3.08
	no	128	23	(1.94-4.84)	(1.95-4.88)
Higher AST	yes	109	29	2.01	2.04
	no	976	521	(1.31-3.06)	(1.33-3.13)
Higher ALT	yes	354	62	3.84	3.79
	no	731	488	(2.84-5.11)	(2.82-5.10)
Hyper-cholesterolemia	yes	645	277	1.45	1.31
	no	440	273	(1.18-1.78)	(1.07-1.81)
Hyper-triglyceridemia	yes	317	49	4.22	4.16
	no	768	501	(3.06-5.18)	(3.01-5.75)
Higher fasting plasma	yes	133	24	3.06	3.11
glucose	no	952	526	(1.96-4.79)	(1.98-4.89)

^1^Aujestment for gender and age

The effect of independent associated risk factors upon NAFLD was examined using the multiple logistic regression model. Table 5 shows that, subsequent to adjustment for confounding factors, the presence of hypertension (yes vs. no, OR = 1.42, 95%CI: 1.02-1.98), higher BMI (yes vs. no, OR = 1.45, 95%CI: 1.38-1.53), hyperuricemia (yes vs. no, OR = 1.79, 95%CI: 1.07-3.00), higher AST (yes vs. no, OR = 2.82, 95%CI: 1.27-4.35), higher ALT (yes vs. no, OR = 2.95, 95%CI: 1.77-3.98), hypertriglyceridemia (yes vs. no, OR = 2.29, 95%CI: 1.60-1.49), and higher FPG (yes vs. no, OR = 2.06, 95%CI: 1.24-3.42) appeared significantly related to NAFLD. Table 5 also shows differences in gender that were revealed by the multiple logistic regression. For males, the significant risk factors related to an NAFLD included the presence of hypertension (yes vs. no, OR = 1.37, 95%CI: 1.04-1.93), a higher BMI (yes vs. no, OR = 1.44, 95%CI: 1.37-1.52), hyperuricemia (yes vs. no, OR = 1.73, 95%CI: 1.07-2.89), higher AST (yes vs. no, OR = 2.70, 95%CI: 1.43-5.00), higher ALT (yes vs. no, OR = 2.83, 95%CI: 1.86-4.30), hypertriglyceridemia (yes vs. no, OR = 2.22, 95%CI: 1.54-3.20), and higher FPG (yes vs. no, OR = 2.23, 95%CI: 1.31-3.79). For females only the presence of a higher BMI (yes vs. no, OR = 1.73, 95%CI: 1.28-2.35) was associated with a risk for NAFLD.

**Table 5:** Multiple logistic regression of associated factors for NAFLD among screened taxi drivers

	Male (n = 1541)		Female (n = 94)		Total(n = 1 635)	
			
	OR	95%CI	OR	95%CI	OR	95%CI
Gender(male vsfemale)	---	---	---	---	1.28	0.78-2.11
Age(yrs)	1.01	0.98-1.02	1.03	0.94-1.13	1.02	0.97-1.04
Hypertension (yes vs no)	1.37	1.04-1.93	2.84	0.45-8.56	1.42	1.02-1.98
Higher BMI(yesvs no)	1.44	1.37-1.52	1.73	1.28-2.35	1.45	1.38-1.53
Hyperuricemia (yes vs no)	1.73	1.07-2.89	---	---	1.79	1.07-3.00
Higher AST (yesvs no)	2.70	1.43-5.00	12.38	0.15-22.04	2.82	1.27-4.35
Higher ALT(yesvs no)	2.83	1.86-4.30	3.23	0.03-4.90	2.95	1.77-3.98
Hypercholesterolemia (yes vs no)	1.10	0.85-1.41	2.37	0.76-7.40	1.16	0.91-1.49
Hypertriglyceridemia(yes vs no)	2.22	1.54-3.20	2.92	0.24-36.07	2.29	1.60-3.28
Higher fasting plasma glucose (yes vs no)	2.23	1.31-3.79	2.70	0.66-4.07	2.06	1.24-3.42

## Discussions

### Prevalence of non-alcoholic fatty liver disease

One of the important benefits of screening programs for NAFLD was that chronic liver disease was often diagnosed as a consequence, as such a screening test was commonly included in the serum chemistry panels conducted on healthy individuals. The relative significance of such results was often ignored when the NAFLD was deemed a slight abnormality The point prevalence of NAFLD varies widely with the population and the diagnosis criteria used[10,11]. Because of this, the prevalence of ultrasound-defined NAFLD in Chinese populations ranged from 1% to more than 30% [11-13]. Previous studies also showed the occupational differences in the prevalence of fatty liver disease. Such differences were highest in administrative officers and white collars workers, followed by laborers, peasants, and monks [11,14]. The prevalence of NAFLD for our study population (66.4%) was much higher than that found in a previous population-based study conducted on the general Chinese populations [11-13]. Taxi drivers face hard work, job stress, and a disruption in normal sleep and waking pattern. An irregular lifestyle, eating habits and sedentary work are also major problems. This might partially explain the high prevalence of NAFLD observed in our study.

### The implications of gender difference as regards associated factors for NAFLD

A detailed analysis of this screening data shows that associated factors for NAFLD in taxi drivers in Taiwan resemble those in other population-based studies [9,11-14]. Our results showed that male gender and an older age are significant risk factors for NAFLD. Such a finding would appear to be consistent with the results of other population-based studies [7,8]. Previously published studies emphasized that NAFLD was more common among females [15], however, recent results have shown that NAFLD may be more prevalent among males [8]. Female hormones have been postulated to protect against NAFLD. This is supported by evidence that NAFLD is twice as common in postmenopausal women as in premenopausal women [8,16]. In addition, NAFLD appears to increase with age. Several academic studies using different diagnostic methods had similar findings [5,12-14].

Our results revealed that a higher FPG was associated with NAFLD. Compared to non diabetic subjects, type 2 diabetics appear to have an increased risk of developing NAFLD, fibrosis, and cirrhosis [17-19]. Among Chinese with NAFLD and without type 2 diabetes in Hong Kong, impaired glucose tolerance without elevation of FPG is common and related to histologically severe liver disease [20,21].

The history of chronic liver disease is usually long and asymptomatic before the development of late-stage disease. The only markers of liver damage during this long phase may be increased serum levels of enzymes such as ALT, AST, and gamma-glutamyltransferase (GGT). Decrease platelet counts and indexes of hepatic biosynthesis usually occur in advanced stages of chronic liver disease [22]. Increasing evidence indicates that significant liver disease may accompany seemingly mild serum aminotransferase level elevations [6]. Consistent with the results of other studies [11], our results revealed that a higher ALT was associated with NAFLD. The determination of the serum ALT level constitutes the most-frequently applied test for the identification of patients suffering from liver disease. This parameter also acts as a surrogate marker for disease severity and/or an index of hepatic activity [23]. The prevalence of fatty liver disease in patients with elevated ALT increased from 26% to 51% in the studied population [11,24]. In mainland China and Taiwan, NAFLD is becoming one of major causes of asymptomatic elevation of liver enzymes [24,25].

Hypertension, higher fasting plasma glucose, and hypertriglyceridemia, were significantly related to NAFLD in this study. The well established term 'metabolic syndrome' remains the most useful and widely accepted description of this cluster of metabolically related cardiovascular risk factors which also predict a high risk of developing diabetes[26]. A long-term follow-up study demonstrated that the presence of metabolic syndrome was associated with an increased risk of NAFLD [27]. Metabolic syndrome can be viewed as a strong predictor of NAFLD and NAFLD can be viewed as a good predictor for the clustering of components of risk factors for metabolic syndrome [21-23]. BMI and waist circumference are also good predictors of NAFLD [11]. In our study, the presence of a higher BMI was associated with a higher risk for NAFLD even after adjusting for other confounding factors. Using the Western criteria for obesity, only 2-3% Asia subjects can be identified as obese [11]. Asians have a higher proportion of visceral fat and a lower proportion of lean body mass than Caucasians with the same BMI condition [28].

In our study, hyperuricemia was also significantly related to males with NAFLD. Clinical studies have shown that a fructose load might lead to a more-substantial increase in serum uric-acid levels among patients suffering chronic hepatitis than in normal subjects [29]. Serum uric-acid levels have been reported elevated among subjects suffering from chronic liver lesions, especially those of a non-infectious origin [6]. Furthermore, the extent of such serum-level elevation appears to be dependent upon the specific severity of the hepatic lesions [6,30]. We were not able to determine the degree of serum uric acid level increase prior to the development of liver disease having developed because of the cross-sectional nature of our study. Further epidemiological and etiological investigations are needed to clarify the pathophysiological mechanisms relating gender differences with uric acid and NAFLD.

### Perceived limitations

One of major limitations of this study was potential selection bias due to the screening of taxi drivers from one area. The potential impact on the prevalence and the study-observed NAFLD-associated risk factors were, in our estimation, inevitable. Nevertheless, given the rather large sample size, the study retained sufficient statistical power to evaluate the presence of any gender differences among the various risk factors for NAFLD. Secondly, because no standard diagnostic elevation has been internationally accepted for defining NAFLD, different studies may elect to set slightly different definitions, such that our estimation of what constituted NAFLD could have suffered from some level of misclassification-bias. This is because ultrasound has limited sensitivity when the degree of steatosis is less than 30% and it is considered highly operator-dependent [8,31]. Thirdly, in consideration of costs, we did not collect information from liver biopsies to confirm, diagnose, and determine the stage of NAFLD. Fourthly, a gender bias may exist since fewer female taxi drivers were a part of our study. Although group sample sizes of 1541 males and 94 females group achieved 97% power to detect difference in NAFLD prevalence between male and females [32,33], a bias estimated is still might have occurred. Finally, our measurements were conducted at only a single point in time and, therefore, may not reflect long-term exposure to important demographic or biochemical factors. The solution to such a quandary would be to conduct a number of prospective longitudinal analogous studies to see if they would complement the community-based (cross-sectional) findings of this study.

## Conclusions

This study found several gender-related dissimilarities regarding the relationship of hyperuricemia, higher AST, higher ALT, hypertriglyceridemia, and higher fasting plasma glucose with NAFLD. Further studies are needed to elucidate the temporal sequence of events that typically lead to NAFLD. Such studies would develop more-satisfactory, non-invasive indicators of liver pathology and assess how gender-related differences are related NAFLD.

## Competing interests

The authors declare that they have no competing interests.

## Authors' contributions

THT, WHC, and JHL carried out the study and drafted the manuscript. THL, HCS, and MHC participated in the design of the study and performed the statistical analysis. THT and JHL conceived of the study, and participated in its design and coordination.

All authors read and approved the final manuscript.
